# SANCDB: a South African natural compound database

**DOI:** 10.1186/s13321-015-0080-8

**Published:** 2015-06-19

**Authors:** Rowan Hatherley, David K Brown, Thommas M Musyoka, David L Penkler, Ngonidzashe Faya, Kevin A Lobb, Özlem Tastan Bishop

**Affiliations:** Department of Biochemistry and Microbiology, Research Unit in Bioinformatics (RUBi), Rhodes University, Grahamstown, South Africa; Department of Chemistry, Rhodes University, Grahamstown, South Africa

**Keywords:** South Africa, Natural products, Chemical databases

## Abstract

**Background:**

Natural products (NPs) are important to the drug discovery process. NP research efforts are expanding world-wide and South Africa is no exception to this. While freely-accessible small molecule databases, containing compounds isolated from indigenous sources, have been established in a number of other countries, there is currently no such online database in South Africa.

**Description:**

The current research presents a South African natural compound database, named SANCDB. This is a curated and fully-referenced database containing compound information for 600 natural products extracted directly from journal articles, book chapters and theses. There is a web interface to the database, which is simple and easy to use, while allowing for compounds to be searched by a number of different criteria. Being fully referenced, each compound page contains links to the original referenced work from which the information was obtained. Further, the website provides a submission pipeline, allowing researchers to deposit compounds from their own research into the database.

**Conclusions:**

SANCDB is currently the only web-based NP database in Africa. It aims to provide a useful resource for the in silico screening of South African NPs for drug discovery purposes. The database is supported by a submission pipeline to allow growth by entries from researchers. As such, we currently present SANCDB the starting point of a platform for a community-driven, curated database to further natural products research in South Africa. SANCDB is freely available at https://sancdb.rubi.ru.ac.za/.

**Electronic supplementary material:**

The online version of this article (doi:10.1186/s13321-015-0080-8) contains supplementary material, which is available to authorized users.

## Background

Natural products (NPs) have been used to treat almost every known ailment, with records from civilizations all over the world, dating as far back as 4,600 years ago [1, 2]. NPs are the result of evolutionary processes that give them a level of structural complexity, chemical diversity, and biological specificity not seen in purely synthetic compounds [3, 4]. NP scaffolds are considered to be crucial to the drug discovery process [4] and between 1981–2010 approximately 64% of approved drugs were derived from or inspired by NPs [5]. Since 2008 there have been 25 newly approved drugs derived from NPs, with 31 additional drugs either at or past phase III clinical trials [6].

A number of NP databases have been developed to assist with in silico drug discovery. These are mostly dominated by compounds extracted from traditional Chinese herbs and medicines. Some of the most well-established being the Traditional Chinese Medicine (TCM) Database@Taiwan [7], CHDD [8] and the Chinese Natural Product Database (CNPD) [9], together totalling just short of 80,000 unique chemical structures [10]. There is also the Encyclopaedia of TCM by Zhou et al. [11], which is a comprehensive literary resource containing structural information and pharmacological data of over 23,000 compounds isolated from nearly 7,000 different plant sources. The TCM applications of different plant sources are also included. The flourishing NP research in Africa has also resulted in a number of similar databases emerging in recent years, though nowhere near the scale of their Chinese counterparts. The most well-established is ConMedNP [12], which contains compounds isolated from plant life used for medicinal purposes in Central Africa. There is currently no database of natural products from Southern Africa, even though this region is rich in both plant and marine life.

South Africa ranks third in the world in terms of its terrestrial biodiversity [13], and it is estimated that 3,000 different plant species are used for medicinal purposes in this country, mostly as part of traditional medicines [14]. Clarkson et al. [15] reported at least moderate antiplasmodial activity from 49% of the 134 different South African medicinal plant extracts tested. Likewise, Bessong et al. [16] reported a series of South African plant extracts that displayed anti-HIV activity. The antimicrobial activity of South African plant life is reviewed by van Vuuren [17], including examples of specific compounds isolated from these plants and their measured antimicrobial activity. Marine NPs are a relatively new source of pharmacological agents [18], but display diverse and unique chemistry [19]. Although early research attempts were made, Southern African marine chemistry research only really took off in the 1990s with compounds isolated showing a great deal of potential as anticancer agents [20].

There is an abundance of information available in literature describing the isolation of compounds from organisms indigenous to South Africa. Whilst found exclusively in literature, these compounds are not easily searchable, especially when attempting to acquire specific information or finding compounds with specific properties. It is difficult to tell which compounds from literature have been included in well-established small-molecule databases, such as ZINC [21, 22] and PubChem [23]. Here we present a database of compounds isolated specifically from South African plants and marine life, which we have developed as a useful resource for NP research.

## Construction and content

### Database design

**Figure 1:**
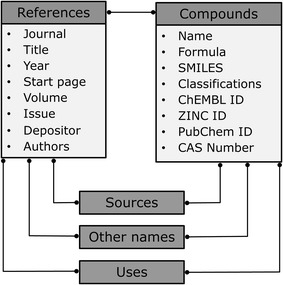
Simplified layout of the SANCDB database schema. Five tables from the database are shown. The *links* between tables represent many-to-many relationships.

SANCDB comprises a MySQL database, integrated into a Django application. The database schema (Figure 1) is mostly centred around the Compounds and References tables. A primary goal of SANCDB was to link all information to at least one referenced work, so this information was always captured before other details were uploaded. Literature searched for included journal articles, theses and textbooks that reported the extraction of compounds from South African plant or marine life. The Rhodes University thesis collection [24] was used to find theses matching these criteria, while PubMed [25] and ScienceDirect [26] were used to find publications that matched these criteria. Additional literature was found using Google [27] and following references to publications and textbooks mentioned in literature that had already found. This applied to compound information, source information and recorded uses for each compound. Sources refer to the organisms from which a given compound was isolated and uses refer to any specific activity tested for a compound; e.g. antimalarial activity. Other names are captured as the name used to refer to a compound in a specific publication, as well as names assigned to the compound by SciFinder [28] if applicable.

### Compound uploading

Compound information was retrieved from literature, including journal articles, theses and book chapters. Information used to populate the remaining four sections of the database was specific to a referenced work and linked to it as such. Since SMILES for a compound are not readily available in publications, the OSRA chemical optical image recognition server [29] was used to generate SMILES for each compound. An image of the compound was sent to OSRA, which had varying degrees of accuracy with image recognition. When using OSRA, the structure produced is shown in a JME molecular editor screen [30] and can be manually edited to fix any obvious errors. Sometimes these are not easy to spot within the editor, so the SMILES of the drawn structure was pasted into Depict [31] to check the structure produced matched the compound image sent to OSRA. Depict provides a quick means to check the compound structure produced by a given SMILES string. If the Depict server was down, the Indigo Toolkit [32] was used to produce the compound image and compared in a similar fashion. Visual inspection did not always ensure errors were detected, so Open Babel’s obprop program was used to produce both the formula and InChI for the compound, based on the SMILES obtained from OSRA. The formula from this SMILES was compared to that reported in literature as an additional means to check for errors. The InChI produced from the SMILES was also checked to find if any chiral centres were not specified. If this was the case, the structure was further inspected to ensure that all known stereocentres (as specified in the original compound image) were indicated when correcting the structure in OSRA. Finally, if a PubChem entry was available for the compound, the InChI from this was compared to that produced from OSRA. Links to external databases were also captured, if available and for published compounds, the CAS registry number was recorded.

### Further data preparation

The SMILES string for each compound was used to prepare a SMILES file for the compound, using Open Babel [33]. The generation of 3D coordinates for compounds was originally also done using Open Babel; however, there are a large number of fused cycloalkanes, which were problematic when generating 3D structures for these molecules, as parts of Open Babel’s 3D generation capabilities are still under development [33]. When converting SMILES of compounds containing these fused rings to 3D structures, Open Babel was unable to retain the stereochemistry at one or more of the bridgehead carbons that formed part of both rings. Since approximately a third of the compounds in database contained at least one fused ring, it was necessary to use CORINA [34, 35] instead to remedy this. The 3D coordinates of each compound were generated using CORINA, saved in SDF format. These were converted to MOL2 and PDB format using Open Babel. Further, compounds were minimized using GAMESS [36] RM1 semi-empirical molecular orbital model. Finally, two-dimensional (2D) images of each compound were produced using the Indigo Toolkit [32]. Open Babel’s obprop program was used to calculate the LogP value for each compound and molecular mass was calculated directly from their formula. The number of hydrogen bond donors and acceptors was calculated from the compound’s SMILES, using Python scripts. The links to external databases used in SANCDB are all based on the entry ID of the compound from that website. The information used to generate the URLs is demonstrated in Additional file 1 and the process managed with Python scripts. A link validator page was also created for depositors. After external IDs were captured, the links were tested by the depositors to ensure that they connected to the correct entry on that site.

### Curation and error checking

Error-finding scripts were written in Python to check for common oversights that may have occurred when uploading compound information. These included entries that were not referenced as depicted in the database layout (Figure 1), as well as references without an author entry or a DOI number. The latter case, which only applied to journal articles, ensured the uniqueness of entries and provided a means to link directly to the article. If a compound could not be converted to a 2D image by Indigo or a 3D structure by CORINA, a flag was raised and the compound SMILES re-inspected. Open Babel’s obprob program was also used to check the SMILES of each compound, as discussed above. A link tester was also incorporated into the depositor’s upload section. While initial entries are uploaded by depositors, these are later checked by a different individual. This involves going back through the literature again, to ensure the correct information was extracted and corrections are made if necessary. Finally, manual spot checks are performed on entries as part of a continuous process.

### Website design

The layout of the website was created by modifying a CSS bootstrap template [37]. The functionality of the site and database queries are controlled mostly by Python scripts through Django, as well as JavaScript and Ajax. A RESTful API is also included using the Django REST framework, documented using the Django REST Swagger API documentation tool.

## Utility and discussion

### Website interface

**Figure 2:**
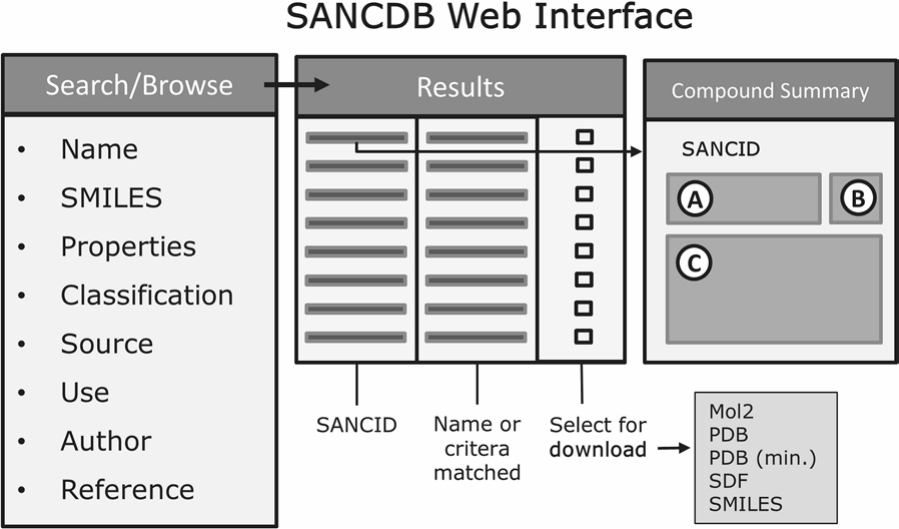
User interface for the search and download of compounds from the SANCDB website. The Search/Browse section represents the different criteria that can be used to query the database. Matching queries are returned on the same page in a tabulated format, displaying the compound’s SANC ID and the criteria matched, as well as a 2D image of the compound (not shown here). Compounds can be selected for download from this page in one of the chemical formats shown. The SANC ID of each compound links to a summary page, divided as follows. *A* Basic compound information and links to external databases; *B* toggle between compound 2D image and 3D representation; *C* additional compound information and links to references.

**Figure 3:**
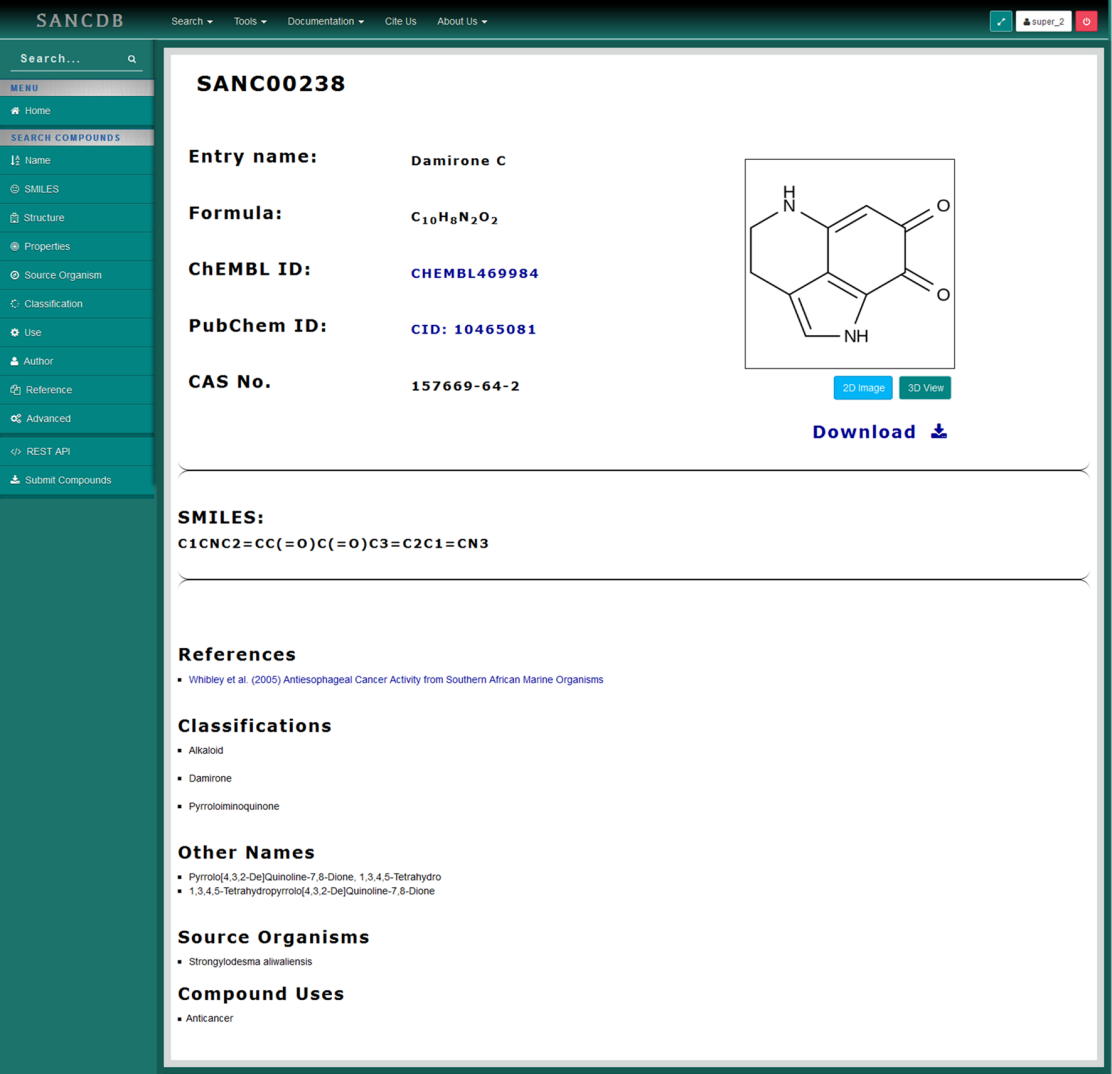
Snapshop of the compound summary page. The interface for the SANCDB website is shown, with a screenshot of the part of the compound summary page for entry SANC00238, as explained in Figure 2.

Users are initially directed to homepage, which contains links to search, browse and download content freely from the website. A representation of the search and download interface is shown in Figure 2. Users can search compounds via the range of criteria listed, and tabulated results are fetched by AJAX requests. This allows users to search, retrieve and download compounds, all from the initial page used for searching without ever having to reload the page. The SANC ID is listed along with a 2D image of the compound, as well as the criteria matched in the search, if applicable. By clicking on the SANC ID for a compound, users will be directed to the compound summary page (Figure 3). This contains all information collected for the compound, including links to ChEMBL [38], DrugBank [39], PubChem [23] and ZINC [21, 22] entries for the compound, if available. Again, a 2D image is shown for the compound and can be switched to a 3D representation, which can be manipulated using the GLmol Molecular Viewer [40]. GLmol is written in JavaScript so it doesn’t require a Java plugin to run, but will only work on modern browsers that support WebGL. References are given for the compound, describing its isolation from South African source organisms. Links are also given to these publications; if a DOI number is available, making them easier to access. The website has been designed so that it is simple and easy to use. A documentation link is provided to help users who are unfamiliar with the website. A REST API has also been developed and a link is given, which shows the API documentation for users who wish to access data from the website without using the web interface. Finally, a link to the SANCDB submission pipeline is provided, which is discussed below. To the best of our knowledge, there is no web-based database containing compounds isolated specifically from Africa. Other African databases, such as ConMedNP [12] do provide compounds and related information, freely available for download; however, only as supplementary data to their publications. The NuBBE database [41] containing natural products from Brazil is probably the closest comparable database to SANCDB. The database also aims to be fully referenced and contains a similar number of compounds. Compounds can be downloaded in one of four different file formats (Mol2, PDB, SDF and SMILES) with the generation of compound 3D coordinates from SMILES done using CORINA. A minimized PDB file can also be downloaded, which is generated using GAMESS [36]. As these are done automatically, the accuracy of the minimized compounds is inspected manually and is an ongoing process.

### SMILES and structure searches

Two of the search pages use compound SMILES as query input. These are the SMILES search page and the structure search page. The only difference between these two pages is that the structure search page incorporates the JSME applet [30], which allows users to draw the structure of the compound they are interested in. Therefore, the user requires no working knowledge of SMILES syntax and can get a visual representation of the desired compound or substructure before searching. The SMILES search page is a quick alternative for searching when the user already has the SMILES of a compound. Both pages will perform a substructure search by default, performed using Open Babel, in order to ensure that the maximum number of relavent structures is returned. A similarity search option will be included in the near future to allow users to find compounds that match the compound SMILES they search for within a specific cut-off value.

### Additional source information

When searching or browsing the source organisms section, the results also include links to a sources page. This contains all information collected about a specific source organism, much like the compounds summary page. Currently included are the compounds isolated from the source organism and references that deal with the source organism. Additionally, the traditional uses of the source organisms themselves are included if available, as mentioned in the reference dealing with the compound isolation. This is captured as a secondary reference and in future this page will be further expanded to include the experimentally tested activity of plant or marine extracts, if available, as well as any other information that may be deemed pertinent. This section of SANCDB may be further enhanced by linking it to additional external resources, such as PlantZAfrica [42]. This resource was developed by the South African National Biodiversity Institute (SANBI) and contains information about plants from across Southern Africa.

### Referenced nature of the database

One of the main aims of our work was to ensure that all information in the database was referenced. Since the database was constructed by manually extracting information from literature we considered it important to keep the links between references and the information retrieved from them. This allows the website to also be used as a research tool, specific to South African natural products. If a user finds a specific compound to be useful, they may follow the links to view literature available on that compound. On a larger scale, the website may be used to find literature describing South African compounds with a particular activity or isolated from a specific organism. This not only makes it easier for a user to find this information, but also increases the exposure of the work done by the researchers who isolated or worked with these compounds. Another benefit to keeping the information fully referenced is enabling users to find compounds within a publication, which is not always a trivial task. A simple example of this would be compound SANC00244, designated the name ‘Autumnalin’, when first isolated [43], but later changed to ‘Eucomnalin’ [44]. In a review by Koorbanally et al. [45], the same compound is referred to as both ‘Autumnalin’ and (E)-5,7-dihydroxy-6-methoxy-3-(4′-hydroxybenzylidene)-4-chromanone. The SANCDB website not only details which publications a compound is found in, but also what names the authors of the publication used when referring to the compound.

### The submission pipeline

As entries are inserted into the database manually, the process requires that we are able to find literature containing this information. Additionally, the isolation of new compounds from natural sources is an on-going process. Consequently, a number of published works might be missed by the depositors at SANCDB. To remedy this, we have developed a submission pipeline to allow researchers to deposit compounds they have isolated or worked with. Thus, SANCDB will provide a platform for a community-driven curated database to further NPs in the country. The submission system mimics the layout of the SANCDB database and submissions will be reviewed by the database curators after each entry is submitted. Users of the system are encouraged to enter as much information about their compound entries as possible. As this is a referenced database, we ask that a reference accompanies each set of compounds submitted. Unpublished work will also be accepted, however, as we aim to develop a non-referenced database alongside SANCDB for these submissions. In this instance, the compounds will be linked to the depositor’s user account, instead of literature. The submission pipeline will allow the community to quickly and easily publicise compounds isolated or worked on by their research groups. This will grant more exposure to their work, as the database will link directly to their publications. This community-driven supplement to the SANCDB website will expand the knowledge base of NP research in South Africa.

### Summary of content

**Figure 4:**
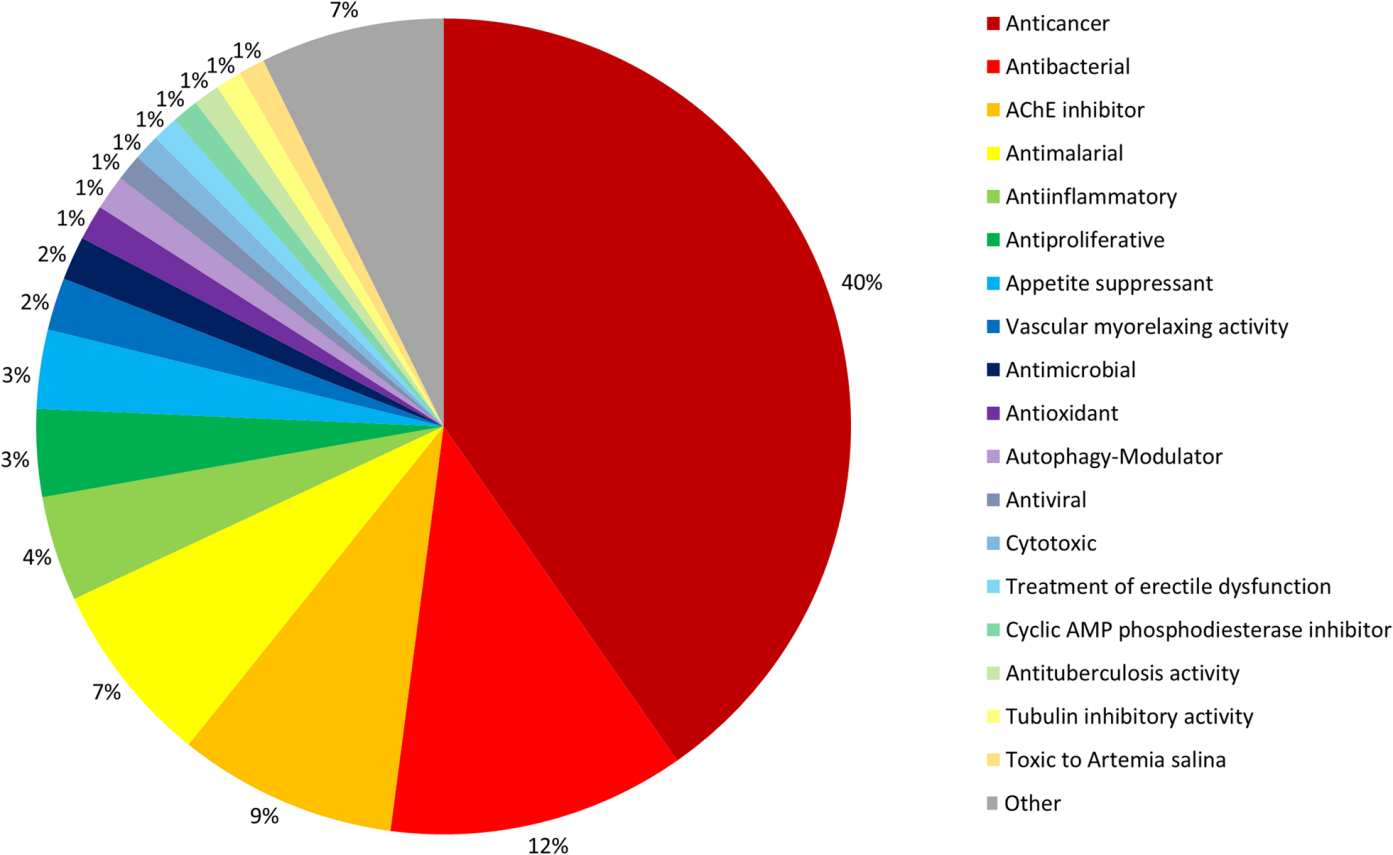
Compound uses within SANCDB. *Chart* showing the distribution of the 284 entries for tested uses of compounds within the database. Uses that comprised less than 1% of entries are grouped in the section labelled ‘Other’.

**Figure 5:**
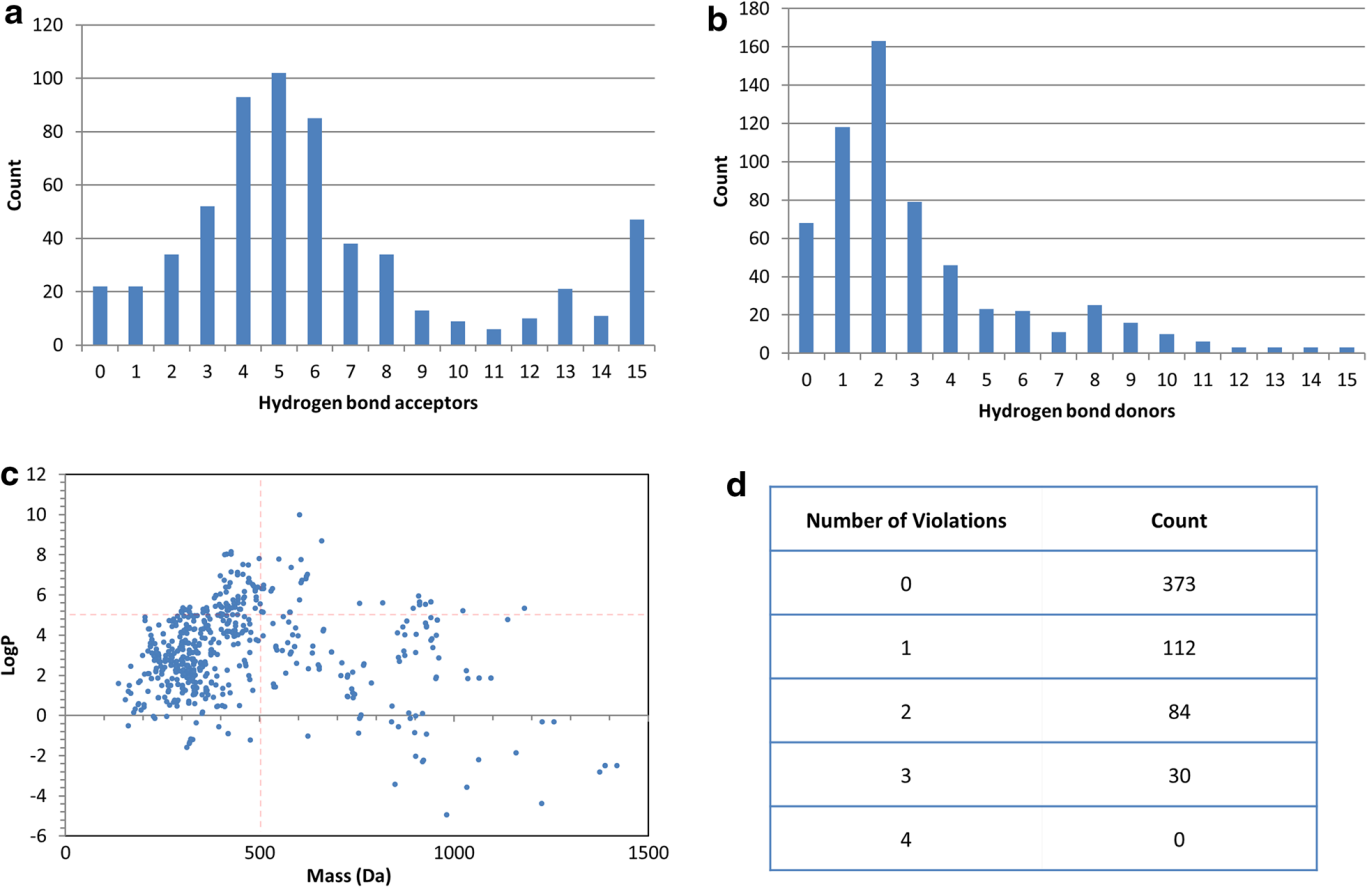
Physicochemical properties of compounds in the database, based on Lipinski’s “rule of five”. **a** Bar graph of compounds grouped by number of hydrogen bond acceptors; **b** bar graph of compounds grouped by number of hydrogen bond donors; **c** scatter plot of the LogP of each compound, plotted against their mass. The *red dotted lines* indicate the cut-off Lipinski values for each axis (LogP ≤5 and mass ≤500 Da); **d** tabulated grouping of compounds in the database with their number of violations of Lipinski’s “rule of five”.

The database currently contains 600 compounds isolated from 143 different South African source organisms. Of the 170 different references used when constructing the database, 164 of them were journal articles, five were from theses and a single entry from a book chapter. A break down of articles by journal and year is shown in Additional file 2 and the list of references is presented in Additional file 3. There are 284 ‘Use’ entries for compounds in the database, 40% of which describe compounds that display some form of anticancer activity (Figure 4). Other common established uses for compounds in the database include antibacterial activity (12%), acetylcholinesterase inhibitory activity (9%) and antimalarial activity (7%). The chemical diversity of the database is further demonstrated by the structural classifications present. Additional file 4(A) indicates the most common classifications present in specific source organisms at a species or genus level. Some of the more prominent structural classifications were linked to specific uses, as indicated in Additional file 4(B). These included anticancer activity (Cephalostatins and Polyketide) and antibacterial activity (Naphthoquinones and Rubrolide Furanones). Lipinski’s rule of five [46] describes four properties of compounds associated with the solubility and permeability of known drugs and is often used to evaluate natural product databases for “drug-likeness” of small molecules [10, 12, 41]. According to the rule, compounds should have no more than five hydrogen-bond donors, no more than ten hydrogen-bond acceptors, have a calculated LogP of no greater than five and a molecular mass of no more than 500 Da. A summary of these properties for compounds in SANCDB is presented in Figure 5. A similar distribution of properties is seen in other natural product databases [10, 12, 41]. More than 60% of compounds in the database violate none of Lipinski’s rules and approximately 80% of compounds violate at most one of the rules. This is a promising indication that compounds in the database have potential to be investigated as prospective drug molecules.

### Structural motifs within the database

In addition to examining the structural classifications of compounds, specific motifs prevalent in the database were also looked at. These were looked at with regard to the recorded activity of compounds in the database, summarised in Additional file 5. It was found that 296 of the 600 compounds in the database contained at least one ether group and that 132 of these compounds were linked to entries in the Uses table. These included compounds with pyranose and furanose rings. Similarly, a large number of compounds with phenol groups were present in the database and also linked to a large number of use entries. The database also contained many compounds with fused ring structures, such as decalin (bicyclo[4.4.0]decane) and hydrindan (bicyclo[4.3.0]nonane), which displayed varying forms of anticancer activity. There was some overlap observed between groups of compounds with specific activity. For example, all 21 compounds in the database with recorded cytotoxic activity against HL-60 leukemia cells contained a pyranose motif, as well as a hydrindan motif. Within this group, were 11 compounds containing a phenol motif, four compounds with a furanose motif and three compounds with a decalin motif.

## Conclusions

We introduce a freely available web-based database containing compounds isolated from South African organisms. This is a referenced database, with a large portion of information linked to literature. SANCDB is simple and easy to use and is currently the only NP database in Africa with a web interface. A variety of different search options are provided to help users find compounds with specific properties or uses, or isolated from certain organisms. The database is constantly growing, with the aid of our submission pipeline, provided to allow researchers to include their own work in this database. Further, it is continually subject to improvement and development. The referenced nature of the database will assist users with keeping track of South African NP literature. Researchers working on isolating NPs can use SANCDB to quickly check if their compounds have been previously found in South African organisms. The SANCDB site is presented as a foundation upon which the knowledge base of NPs in South Africa can be expanded and enhanced by researchers from across the country. This online interface will grant exposure to South African NP research, allowing users to easily find information about compounds isolated from South African organisms, as well as download these for use in virtual screening.

## Availability and requirements

SANCDB is freely available at https://sancdb.rubi.ru.ac.za/.

## Additional files

Additional file 1:
**S-Data 1.** External IDs and URLs for compound SANC00103. The IDs of the compound is shown for ChEMBL, ZINC, PubChem and DrugBank along with the URL that links to that compound entry on each website. In the URL section, the portion of the external ID used in the URL is underlined. Additional file 2:
**S-Data 2.** Number of references by year and by journal. A) The number of references is plotted by year. B) Table indicating the number of references from each journal used. The number of references from theses and book chapters are also displayed. Additional file 3:
**S-Data 3.** List of references included in the SANCDB database. Additional file 4:
**S-Data 4.** Structural classifications of compounds within SANCDB, by source organism and by use. A) The most common compound classifications found in specific source organisms are tabulated. B) The most common compound classifications that were associated with a specific use. Classifications were included in each table if they related to five or more compounds associated with the source or use. Additional file 5:
**S-Data 5.** Structural motifs of compounds within SANCDB. The names and structures of specific motifs present in the compounds in SANCDB are shown. Next to this is the number of compounds in the database which contain the structural motif, as well as the number of these compounds with recorded uses in SANCDB.

## Abbreviations

SANCDB: South African natural compound database
NP: natural product
TCM: Traditional Chinese Medicine
REST: REpresentational State Transfer
API: Application Programming Interface
Ajax: Asynchronous JavaScript and XML
JSME: a free molecule editor in JavaScript
SMILES: Simplified Molecular Input Line Entry System
PDB: Protein Data Bank
2D: two-dimensional
3D: three-dimensional
CSS: cascading style sheets
