# Supplementary material for: SANCDB: a South African natural compound database
Source: J Cheminform. 2015 Jun 19;7:29. doi: 10.1186/s13321-015-0080-8 (PMC4471313; doi:10.1186/s13321-015-0080-8)
Supplement: Additional file 1: — S-Data 1. External IDs and URLs for compound SANC00103. The IDs of the compound is shown for ChEMBL, ZINC, PubChem and DrugBank along with the URL that links to that compound entry on each website. In the URL section, the portion of the external ID used in the URL is underlined. [file 13321_2015_80_MOESM1_ESM.docx]

| **Site** | **External ID** | **URL** |
| --- | --- | --- |
| ChEMBL | CHEMBL130415 | https://www.ebi.ac.uk/chembldb/index.php/compound/inspect/CHEMBL130415 |
| ZINC | ZINC03870339 | http://zinc.docking.org/substance/ZINC03870339 |
| PubChem | CID: 10425234 | http://pubchem.ncbi.nlm.nih.gov/summary/summary.cgi?cid=10425234 |
| DrugBank | DB03823 | http://www.drugbank.ca/drugs/DB03823 |
