# Supplementary material for: SANCDB: a South African natural compound database
Source: J Cheminform. 2015 Jun 19;7:29. doi: 10.1186/s13321-015-0080-8 (PMC4471313; doi:10.1186/s13321-015-0080-8)
Supplement: Additional file 2: — S-Data 2. Number of references by year and by journal. A) The number of references is plotted by year. B) Table indicating the number of references from each journal used. The number of references from theses and book chapters are also displayed. [file 13321_2015_80_MOESM2_ESM.docx]

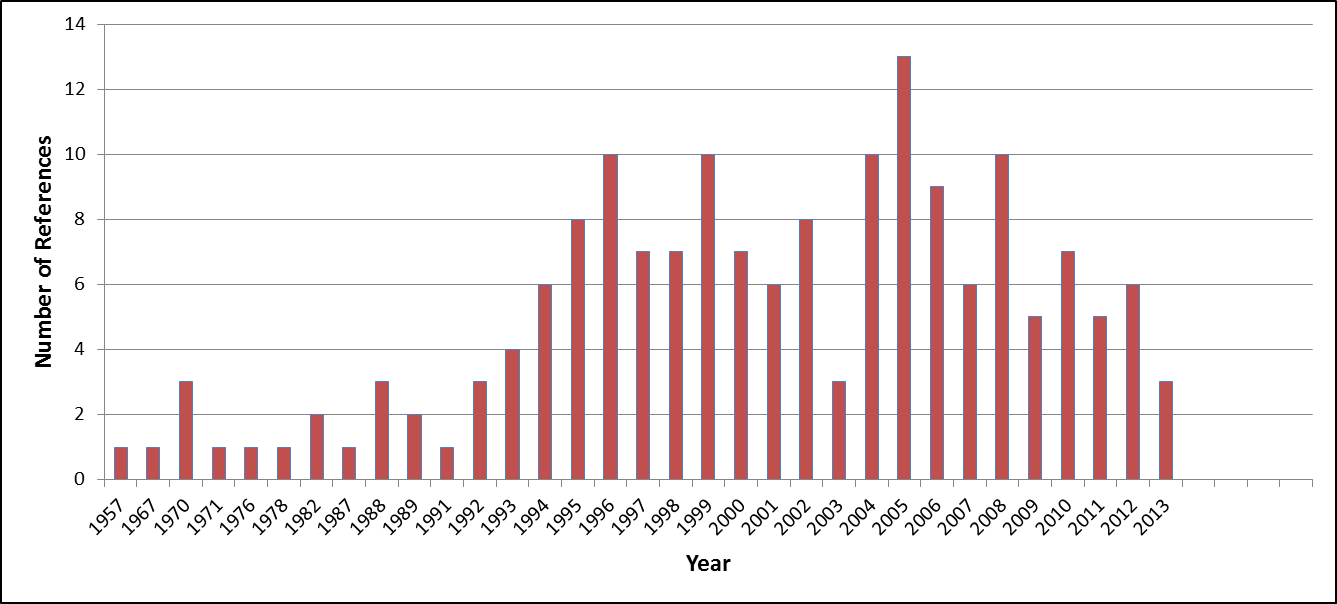


| **Journal** | **Count** |
| --- | --- |
| Journal of Natural Products | 49 |
| Phytochemistry | 32 |
| Journal of Ethnopharmacology | 13 |
| Tetrahedron Letters | 9 |
| Tetrahedron | 9 |
| Biochemical Systematics and Ecology | 6 |
| South African Journal of Botany | 5 |
| Theses | 5 |
| Bioorganic & Medicinal Chemistry Letters | 4 |
| Canadian Journal of Chemistry | 4 |
| South African Journal of Chemistry | 3 |
| Chemical Communications | 2 |
| Helvetica Chimica Acta | 2 |
| Journal of the American Chemical Society | 2 |
| Natural Product Research | 2 |
| South African Journal of Science | 2 |
| The Journal of Organic Chemistry | 2 |
| Annals of the New York Academy of Sciences | 1 |
| Bioorganic & Medicinal Chemistry | 1 |
| Book Chapters | 1 |
| Cancer Cell International | 1 |
| Chemical and Pharmaceutical Bulletin | 1 |
| Experientia | 1 |
| Fitoterapia | 1 |
| Journal of Medicinal Chemistry | 1 |
| Journal of Pharmacy and Pharmacology | 1 |
| Journal of the Chemical Society | 1 |
| Life Sciences | 1 |
| Molecules | 1 |
| Narurforsch | 1 |
| Natural Product Letters | 1 |
| Organic Letters | 1 |
| Phytochemical communication | 1 |
| Plant Medica | 1 |
| Steroids | 1 |
| The Journal of Biological Chemistry | 1 |
