# Supplementary material for: SANCDB: a South African natural compound database
Source: J Cheminform. 2015 Jun 19;7:29. doi: 10.1186/s13321-015-0080-8 (PMC4471313; doi:10.1186/s13321-015-0080-8)
Supplement: Additional file 3: — S-Data 3. List of references included in the SANCDB database. [file 13321_2015_80_MOESM3_ESM.docx]

1. Adelekan AM, Prozesky EA, Hussein AA, Ureña LD, Van Rooyen PH, Liles DC, Meyer JJM, Rodríguez B: **Bioactive diterpenes and other constituents of Croton steenkampianus**. *J Nat Prod* 2008, **71**:1919–1922.

2. Aderogba MA, Kgatle DT, McGaw LJ, Eloff JN: **Isolation of antioxidant constituents from Combretum apiculatum subsp. apiculatum**. *South African J Bot* 2012, **79**:125–131.

3. Afolayan AF, Mann MGA, Lategan CA, Smith PJ, Bolton JJ, Beukes DR: **Antiplasmodial halogenated monoterpenes from the marine red alga Plocamium cornutum**. *Phytochemistry* 2009, **70**:597–600.

4. Afolayan AF, Bolton JJ, Lategan CA, Smith PJ, Beukes DR: **Fucoxanthin, tetraprenylated toluquinone and toluhydroquinone metabolites from Sargassum heterophyllum inhibit the in vitro growth of the malaria parasite Plasmodium falciparum**. *Zeitschrift fur Naturforsch - Sect C J Biosci* 2008, **63**:848–852.

5. Liddell JR, Logie CG: **7-Angelyl-1-methylenepyrrolizidines from Senecio chrysocoma**. *Phytochemistry* 1993, **34**:1198–1199.

6. Antunes EM: **Pyrroloiminoquinone metabolites from South African latrunculid sponges**. Rhodes University; 2003.

7. Antunes EM, Afolayan AF, Chiwakata MT, Fakee J, Knott MG, Whibley CE, Hendricks DT, Bolton JJ, Beukes DR: **Identification and in vitro anti-esophageal cancer activity of a series of halogenated monoterpenes isolated from the South African seaweeds Plocamium suhrii and Plocamium cornutum**. *Phytochemistry* 2011, **72**:769–772.

8. Antunes EM, Copp BR, Davies-Coleman MT, Samaai T: **Pyrroloiminoquinone and related metabolites from marine sponges.** *Nat Prod Rep* 2005, **22**:62–72.

9. Ayers S, Zink DL, Mohn K, Powell JS, Brown CM, Murphy T, Brand R, Pretorius S, Stevenson D, Thompson D, Singh SB: **Scutiaquinones A and B, perylenequinones from the roots of scutia myrtina with anthelmintic activity**. *J Nat Prod* 2007, **70**(Figure 1):425–427.

10. Bangani V, Crouch NR, Mulholland DA: **Homoisoflavanones and stilbenoids from Scilla nervosa**. *Phytochemistry* 1999, **51**:947–951.

11. Bastida Armengol J, Berkov S, Torras Clavería L, Pigni NB, Andradre JP De, Martínez V, Codina Mahrer C, Viladomat Meya F: *Chemical and Biological Aspects of Amaryllidaceae Alkaloids*. *Volume 661*; 2011:65–100.

12. Bessong PO, Obi CL, Andréola M-L, Rojas LB, Pouységu L, Igumbor E, Meyer JJM, Quideau S, Litvak S: **Evaluation of selected South African medicinal plants for inhibitory properties against human immunodeficiency virus type 1 reverse transcriptase and integrase.** *J Ethnopharmacol* 2005, **99**:83–91.

13. Beukes DR, Davies-Coleman MT, Kelly-Borges M, Harper MK, Faulkner DJ: **Dilemmaones A-C, unusual indole alkaloids from a mixed collection of South African sponges**. *J Nat Prod* 1998, **61**:699–701.

14. Beukes DR: **Structural and synthetic investigations of South African marine natural products**. Rhodes University; 1999.

15. Beukes DR, Davies-Coleman MT: **Novel polypropionates from the South African marine mollusc Siphonaria capensis**. *Tetrahedron* 1999, **55**:4051–4056.

16. Beukes DR, Davies-Coleman MT, Eggleston DS, Haltiwanger RC, Tomkowicz B: **New polyhydroxylated pregnadienes from the South African soft coral Pieterfaurea unilobata**. *J Nat Prod* 1997, **60**:573–577.

17. Böhler P, Tamm C: **The homo-isoflavones, a new class of natural product. Isolation and structure of eucomin and eucomol.** *Tetrahedron Lett* 1967, **8**:3479–3483.

18. Bohlmann F, Zdero C: **New sesquiterpenes from Senecio oxyodontus**. *Phytochemistry* 1978, **17**:1591–1593.

19. Bolton JJ, Davies-Coleman MT, Coyne VE: **Innovative processes and products involving marine organisms in South Africa**. *African J Mar Sci* 2013, **35**(July 2014):449–464.

20. Brecknell DJ, Collett LA, Davies-Coleman MT, Garson MJ, Jones DD: **New non-contiguous polypropionates from marine molluscs: A comment on their natural product status**. *Tetrahedron* 2000, **56**:2497–2502.

21. Brine ND, Campbell WE, Bastida J, Herrera MR, Viladomat F, Codina C, Smith PJ: **A dinitrogenous alkaloid from Cyrtanthus obliquus**. *Phytochemistry* 2002, **61**:443–447.

22. Bromley CL, Parker-Nance S, de la Mare J-A, Edkins AL, Beukes DR, Davies-Coleman MT: **Halogenated Oxindole and Indoles from the South African Marine Ascidian Distaplia skoogi**. *South African J Chem* 2013, **66**:64–68.

23. Bromley CL, Popplewell WL, Pinchuck SC, Hodgson AN, Davies-Coleman MT: **Polypropionates from the South African marine mollusk Siphonaria oculus.** *J Nat Prod* 2012, **75**:497–501.

24. Hirano T, Oka K, Mimaki Y, Kuroda M, Sashida Y: **Potent growth inhibitory activity of a novel Ornithogalum cholestane glycoside on human cells: Induction of apoptosis in promyelocytic leukemia HL-60 cells**. *Life Sci* 1996, **58**:789–798.

25. Cheesman L, Nair JJ, Van Staden J: **Antibacterial activity of crinane alkaloids from Boophone disticha (Amaryllidaceae)**. *J Ethnopharmacol* 2012, **140**:405–408.

26. Chukwujekwu JC, Lategan CA, Smith PJ, Van Heerden FR, Van Staden J: **Antiplasmodial and cytotoxic activity of isolated sesquiterpene lactones from the acetone leaf extract of Vernonia colorata**. *South African J Bot* 2009, **75**:176–179.

27. Chukwujekwu JC, Smith P, Coombes PH, Mulholland DA, Van Staden J: **Antiplasmodial diterpenoid from the leaves of Hyptis suaveolens**. *J Ethnopharmacol* 2005, **102**:295–297.

28. Pettit GR, Kamano Y, Inoue M, Dufresne C, Boyd MR, Herald CL, Schmidt JM, Doubek DL, Christie ND: **Antineoplastic agents. 214. Isolation and structure of cephalostatins 7-9**. *J Org Chem* 1992, **57**:429–431.

29. Copley RCB, Davies-Coleman MT, Edmonds DR, Faulkner DJ, McPhail KL: **Absolute stereochemistry of ibhayinol from a South African sea hare**. *J Nat Prod* 2002, **65**:580–582.

30. Corr LT, Sealy JC, Horton MC, Evershed RP: **A novel marine dietary indicator utilising compound-specific bone collagen amino acid ??13C values of ancient humans**. *J Archaeol Sci* 2005, **32**:321–330.

31. Dagne E, Bisrat D, Van Wyk BE, Viljoen AM: **10-hydroxyaloin B 6’-O-acetate, an oxanthrone from Aloe claviflora**. *J Nat Prod* 1998, **61**:256–257.

32. Dai J, Fishback JA, Zhou YD, Nagle DG: **Sodwanone and yardenone triterpenes from a South African species of the marine sponge Axinella inhibit hypoxia-inducible factor-1 (HIF-1) activation in both breast and prostate tumor cells**. *J Nat Prod* 2006, **69**(Table 1):1715–1720.

33. Davidson BS, Molinski TF, Barrows LR, Ireland CM: **Varacin: A Novel Benzopentathiepin from Lissoclinum vareau That Is Cytotoxic toward a Human Colon Tumor**. *J Am Chem Soc* 1991, **113**:4709–4710.

34. Davies-Coleman MT, Beukes DR: **Ten years of marine natural products research at Rhodes University**. *S Afr J Sci* 2004, **100**(December):539–544.

35. Davies-Coleman MT, Gray CA, Schleyer MH: **Cembrane diterpenes from the Southern African soft coral Cladiella kashmani**. *J Nat Prod* 2000, **63**(Table 1):1551–1553.

36. De La Mare JA, Lawson JC, Chiwakata MT, Beukes DR, Edkins AL, Blatch GL: **Quinones and halogenated monoterpenes of algal origin show anti-proliferative effects against breast cancer cells in vitro**. *Invest New Drugs* 2012, **30**:2187–2200.

37. De la Mare J-A, Sterrenberg JN, Sukhthankar MG, Chiwakata MT, Beukes DR, Blatch GL, Edkins AL: **Assessment of potential anti-cancer stem cell activity of marine algal compounds using an in vitro mammosphere assay.** *Cancer Cell Int* 2013, **13**:39.

38. De Wet H, Van Heerden FR, Van Wyk BE: **Alkaloidal variation in Cissampelos capensis (Menispermaceae)**. *Molecules* 2011, **16**:3001–3009.

39. Clarkson C, Stærk D, Hansen SH, Smith PJ, Jaroszewski JW: **Discovering new natural products directly from crude extracts by HPLC-SPE-NMR: Chinane diterpenes in Harpagophytum procumbens**. *J Nat Prod* 2006, **69**:527–530.

40. Drewes SE, Horn MM, Khan F, Munro OQ, Dhlamini JTB, Rakuambo C, Marion Meyer JJ: **Minor pyrano-isoflavones from Eriosema kraussianum: Activity-, structure-, and chemical reaction studies**. *Phytochemistry* 2004, **65**:1955–1961.

41. Drewes SE, Horn MM, Munro OQ, Dhlamini JTB, Meyer JJM, Rakuambo NC: **Pyrano-isoflavones with erectile-dysfunction activity from Eriosema kraussianum**. *Phytochemistry* 2002, **59**:739–747.

42. Eldeen IMS, Van Heerden FR, Van Staden J: **In vitro biological activities of niloticane, a new bioactive cassane diterpene from the bark of Acacia nilotica subsp. kraussiana**. *J Ethnopharmacol* 2010, **128**:555–560.

43. Elgorashi EE, Stafford GI, Van Staden J: **Acetylcholinesterase enzyme inhibitory effects of Amaryllidaceae alkaloids**. *Planta Med* 2004, **70**:260–262.

44. Erasto P, Grierson DS, Afolayan AJ: **Bioactive sesquiterpene lactones from the leaves of Vernonia amygdalina**. *J Ethnopharmacol* 2006, **106**:117–120.

45. Fennell CW, Elgorashi EE, van Staden J: **Alkaloid production in Crinum moorei cultures.** *J Nat Prod* 2003, **66**:1524–1526.

46. Pettit GR, Xu J, Schmidt JM, Boyd MR: **Isolation and structure of the exceptional Pterobranchia human cancer inhibitors cephalostatins 16 and 171**. *Bioorg Med Chem Lett* 1995, **5**:2027–2032.

47. Finckh RE, Tamm C: **The homo-isoflavones III. isolation and structure of punctatin, 3,9-dihydro-punctatin, 4’-O-methyl-3,9-dihydro-punctatin, 4'-demethyl-eucomin and 4'-demethyl-5-O-methyl-3,9-dihydro-eucomin**. *Experientia* 1970, **26**:472–473.

48. Fouche G, Cragg GM, Pillay P, Kolesnikova N, Maharaj VJ, Senabe J: **In vitro anticancer screening of South African plants**. *J Ethnopharmacol* 2008, **119**:455–461.

49. Fu X, Li XC, Smillie TJ, Carvalho P, Mabusela W, Syce J, Johnson Q, Folk W, Avery MA, Khan IA: **Cycloartane glycosides from Sutherlandia frutescens**. *J Nat Prod* 2008, **71**:1749–1753.

50. Fusi F, Ferrara A, Koorbanally C, Crouch NR, Mulholland DA, Sgaragli G: **Vascular myorelaxing activity of isolates from South African Hyacinthaceae partly mediated by activation of soluble guanylyl cyclase in rat aortic ring preparations.** *J Pharm Pharmacol* 2008, **60**:489–497.

51. Gray CA, Davies-Coleman MT, McQuaid C: **Labdane diterpenes From the South African marine pulmonate Trimusculus costatus**. *Nat Prod Lett* 1998, **12**(June 2014):47–53.

52. Guthrie-strachan JJ: **Chemical Studies of Necic Acid Analogues**. Rhodes University; 1996.

53. Viladomat F, Bastida J, Codina C, Campbell WE, Mathee S: **Alkaloids from Brunsvigia josephinæ**. *Phytochemistry* 1994, **35**:809–812.

54. Heinrich MR, Steglich W: **Synthesis of (-)-(3S)-1-(3-aminopropyl)-3-methylazacyclodecane, the structure proposed for the marine alkaloid haliclorensin**. *Tetrahedron Lett* 2001, **42**:3287–3289.

55. Herrera MR, Machocho AK, Nair JJ, Campbell WE, Brun R, Viladomat F, Codina C, Bastida J: **Alkaloids from Cyrtanthus elatus**. *Fitoterapia* 2001, **72**:444–448.

56. Hooper GJ: **Biologically active natural products from south african marine invertebrates**. Rhodes University; 1996.

57. Hooper GJ, Davies-Coleman MT: **New Metabolites from the South African soft coral Capnella thyrsoidea**. *Tetrahedron* 1995, **51**:9973–9984.

58. Hooper GJ, Davies-Coleman MT: **Sesquiterpene hydroquinones from the South African soft coral Alcyonium fauri**. *Tetrahedron Lett* 1995, **36**:3265–3268.

59. Hooper GJ, Davies-coleman MT, Coetzee PS: **Amines from a South African Marine Ascidian**. *Nat Prod Lett* 1995, **6**(July 2014):31–35.

60. Hooper GJ, Davies-Coleman MT, Kelly-Borges M, Coetzee PS: **New alkaloids from a South African latrunculid sponge**. *Tetrahedron Lett* 1996, **37**:7135–7138.

61. Hooper GJ, Davies-Coleman MT, Schleyer M: **New diterpenes from the South African soft coral Eleutherobia aurea**. *J Nat Prod* 1997, **60**:889–893.

62. Howell H, Malan E, Steenkamp JA, Brandt EV, Brand J: **Identification of two novel promelacacinidin dimers from Acacia nigrescens**. *J Nat Prod* 2002, **65**(C):769–771.

63. Hussein AA, Meyer JJM, Jimeno ML, Rodríguez B: **Bioactive diterpenes from Orthosiphon labiatus and Salvia africana-lutea**. *J Nat Prod* 2007, **70**:293–295.

64. Jakupovic J, Zdero C, Grenz M, Tsichritzis F, Lehmann L, Hashemi-Nejad SM, Bohlmann F: **Twenty-one acylphloroglucinol derivatives and further constituents from south african Helichrysum species**. *Phytochemistry* 1989, **28**:1119–1131.

65. Kamdem Waffo AF, Coombes PH, Crouch NR, Mulholland DA, El Amin SMM, Smith PJ: **Acridone and furoquinoline alkaloids from Teclea gerrardii (Rutaceae: Toddalioideae) of southern Africa**. *Phytochemistry* 2007, **68**:663–667.

66. Kashman Y, Koren-Goldshlager G, Gravalos MDG, Schleyer M: **Halitulin, a new cytotoxic alkaloid from the marine sponge Haliclona tulearensis**. *Tetrahedron Lett* 1999, **40**:997–1000.

67. Ketzinel S, Rudi A, Schleyer M, Benayahu Y, Kashman Y: **Sarcodictyin A and two novel diterpenoid glycosides, eleuthosides A and B, from the soft coral Eleutherobia aurea**. *J Nat Prod* 1996, **59**:873–875.

68. Keyzers RA, Arendse CE, Hendricks DT, Samaai T, Davies-Coleman MT: **Makaluvic acids from the South African latrunculid sponge Strongylodesma aliwaliensis**. *J Nat Prod* 2005, **68**:506–510.

69. Keyzers RA, Daoust J, Davies-Coleman MT, Van Soest R, Balgi R, Donohue E, Roberge M, Andersen RJ: **Autophagy-modulating aminosteroids isolated from the sponge Cliona celata**. *Org Lett* 2008, **10**:2959–2962.

70. Keyzers RA, Samaai T, Davies-Coleman MT: **Novel pyrroloquinoline ribosides from the South African latrunculid sponge Strongylodesma aliwaliensis**. *Tetrahedron Lett* 2004, **45**:9415–9418.

71. Knott MG, Mkwananzi H, Arendse CE, Hendricks DT, Bolton JJ, Beukes DR: **Plocoralides A-C, polyhalogenated monoterpenes from the marine alga Plocamium corallorhiza**. *Phytochemistry* 2005, **66**:1108–1112.

72. Koorbanally C, Mulholland DA, Crouch NR: **Eudesmane-type sesquiterpenoids from Urginea epigea (Urgineoideae; Hyacinthaceae)**. *Biochem Syst Ecol* 2005, **33**:295–299.

73. Koorbanally NA, Crouch NR, Harilal A, Pillay B, Mulholland DA: **Coincident isolation of a novel homoisoflavonoid from Resnova humifusa and Eucomis montana (Hyacinthoideae: Hyacinthaceae)**. *Biochem Syst Ecol* 2006, **34**:114–118.

74. Koorbanally NA, Koorbanally C, Harilal A, Mulholland DA, Crouch NR: **Bufadienolides from Drimia robusta and Urginea epigea (Hyacinthaceae)**. *Phytochemistry* 2004, **65**:3069–3073.

75. Koorbanally NA, Randrianarivelojosia M, Mulholland DA, Van Ufford LQ, Van den Berg AJJ: **Bioactive constituents of Cedrelopsis microfoliata**. *J Nat Prod* 2002, **65**:1349–1352.

76. Koren-Goldshlager G, Kashman Y, Schleyer M: **Haliclorensin, a novel diamino alkaloid from the marine sponge Haliclona tulearensis**. *J Nat Prod* 1998, **61**:282–284.

77. Koren-Goldshlager G, Klein P, Rudi A, Benayahu Y, Schleyer M, Kashman Y: **Sindurol and nephthoside: New tetraprenyltoluquinols from the soft coral Sinularia dura and Nephthea sp.** *J Nat Prod* 1996, **59**:262–266.

78. Kubo S, Mimaki Y, Terao M, Sashida Y, Nikaido T, Ohmoto T: **Acylated cholestane glycosides from the bulbs of Ornithogalum saundersiae**. *Phytochemistry* 1992, **31**:3969–3973.

79. Kuete V, Ngameni B, Mbaveng AT, Ngadjui B, Meyer JJM, Lall N: **Evaluation of flavonoids from Dorstenia barteri for their antimycobacterial, antigonorrheal and anti-reverse transcriptase activities**. *Acta Trop* 2010, **116**:100–104.

80. Kuete V, Tangmouo JG, Marion Meyer JJ, Lall N: **Diospyrone, crassiflorone and plumbagin: three antimycobacterial and antigonorrhoeal naphthoquinones from two Diospyros spp.** *Int J Antimicrob Agents* 2009, **34**:322–325.

81. Kuete V, Wabo GF, Ngameni B, Mbaveng AT, Metuno R, Etoa FX, Ngadjui BT, Beng VP, Meyer JJM, Lall N: **Antimicrobial activity of the methanolic extract, fractions and compounds from the stem bark of Irvingia gabonensis (Ixonanthaceae)**. *J Ethnopharmacol* 2007, **114**:54–60.

82. Kuroda M, Mimaki Y, Yokosuka A, Sashida Y: **Cholestane glycosides from the bulbs of Galtonia candicans and their cytotoxicity.** *Chem Pharm Bull (Tokyo)* 2001, **49**:1042–1046.

83. Kuroda M, Mimaki Y, Sashida Y, Nikaido T, Ohmoto T: **Structure of a novel 22-homo-23-norcholestane trisaccharide from Ornithogalum saundersiae**. *Tetrahedron Lett* 1993, **34**:6073–6076.

84. Kuroda M, Mimaki Y, Ori K, Sakagami H, Sashida Y: **Steroidal glycosides from the bulbs of Ornithogalum thyrsoides**. *J Nat Prod* 2004, **67**:1690–1696.

85. Kuroda M, Mimaki Y, Sashida Y: **Cholestane rhamnosides from the bulbs of Ornithogalum saundersiae**. *Phytochemistry* 1999, **52**:445–452.

86. Kuroda M, Mimaki Y, Sashida Y: **Saundersiosides C-H, rearranged cholestane glycosides from the bulbs of Ornithogalum saundersiae and their cytostatic activity on HL-60 cells**. *Phytochemistry* 1999, **52**:435–443.

87. Kuroda M, Mimaki Y, Sashida Y, Hirano T, Oka K, Dobashi A: **Novel cholestane glycosides from the bulbs of Ornithogalum saundersiae and their cytostatic activity on leukemia HL-60 and MOLT-4 cells**. *Tetrahedron* 1997, **53**:11549–11562.

88. Kuroda M, Mimaki Y, Yokosuka A, Hasegawa F, Sashida Y: **Cholestane glycosides from the bulbs of Ornithogalum thyrsoides and their cytotoxic activity against HL-60 leukemia cells**. *J Nat Prod* 2002, **65**:1417–1423.

89. Kuroda M, Mimaki Y, Yokosuka A, Sashida Y, Beutler JA: **Cytotoxic cholestane glycosides from the bulbs of Ornithogalum saundersiae**. *J Nat Prod* 2001, **64**:88–91.

90. Kuroda M, Ori K, Mimaki Y: **Ornithosaponins A-D, four new polyoxygenated steroidal glycosides from the bulbs of Ornithogalum thyrsoides**. *Steroids* 2006, **71**:199–205.

91. Langat MK, Crouch NR, Smith PJ, Mulholland DA: **Cembranolides from the leaves of croton gratissimus**. *J Nat Prod* 2011, **74**:2349–2355.

92. Langlois A, Mulholland DA, Duncan GD, Crouch NR, Edwards TJ: **A novel 3-benzylchromone from the South African Lachenalia rubida (Hyacinthaceae)**. *Biochem Syst Ecol* 2005, **33**:961–966.

93. Lategan CA, Campbell WE, Seaman T, Smith PJ: **The bioactivity of novel furanoterpenoids isolated from Siphonochilus aethiopicus**. *J Ethnopharmacol* 2009, **121**:92–97.

94. Li XC, Van der Bijl P, Wu CD: **Binaphthalenone glycosides from African chewing sticks, Diospyros lycioides**. *J Nat Prod* 1998, **61**(Table 1):817–820.

95. Liddell JR, Logie CG: **A re-investigation of the alkaloids of Senecio pterophorus**. *Phytochemistry* 1993, **34**:1629–1631.

96. Logie CG: **The Pyrrolizidine Alkaloids of Senecio Chrysocoma and Senecio Paniculatus**. Rhodes University; 1995.

97. Mann MGA, Mkwananzi HB, Antunes EM, Whibley CE, Hendricks DT, Bolton JJ, Beukes DR: **Halogenated monoterpene aldehydes from the South African marine alga Plocamium corallorhiza**. *J Nat Prod* 2007, **70**:596–599.

98. Mathabe MC, Hussein AA, Nikolova R V., Basson AE, Meyer JJM, Lall N: **Antibacterial activities and cytotoxicity of terpenoids isolated from Spirostachys africana**. *J Ethnopharmacol* 2008, **116**:194–197.

99. Mativandlela SPN, Muthivhi T, Kikuchi H, Oshima Y, Hamilton C, Hussein AA, Van Der Walt ML, Houghton PJ, Lall N: **Antimycobacterial flavonoids from the leaf extract of Galenia africana**. *J Nat Prod* 2009, **72**:2169–2171.

100. Matochko WL, James A, Lam CW, Kozera DJ, Ata A, Gengan RM: **Triterpenoidal alkaloids from buxus natalensis and their acetylcholinesterase inhibitory activity**. *J Nat Prod* 2010, **73**(Figure 1):1858–1862.

101. Mbaveng AT, Ngameni B, Kuete V, Simo IK, Ambassa P, Roy R, Bezabih M, Etoa FX, Ngadjui BT, Abegaz BM, Meyer JJM, Lall N, Beng VP: **Antimicrobial activity of the crude extracts and five flavonoids from the twigs of Dorstenia barteri (Moraceae)**. *J Ethnopharmacol* 2008, **116**:483–489.

102. McPhail KL, Davies-Coleman MT, Starmer J: **Sequestered chemistry of the Arminacean nudibranch Leminda millecra in Algoa Bay, South Africa**. *J Nat Prod* 2001, **64**:1183–1190.

103. McPhail KL, Davies-Coleman MT, Copley RCB, Eggleston DS: **New halogenated sesquiterpenes from South African specimens of the circumtropical sea hare Aplysia dactylomela**. *J Nat Prod* 1999, **62**:1618–1623.

104. McPhail KL, Rivett DEA, Lack DE, Davies-Coleman MT: **The Structure and Synthesis of Tsitsikammafuran: A New Furanosesquiterpene from a South African Dysidea Sponge**. *Tetrahedron* 2000, **56**:9391–9396.

105. McPhail K, Davies-Coleman MT, Coetzee P: **A new furanosesterterpene from the South African nudibranch Hypselodoris capensis and a Dictyoceratida sponge**. *J Nat Prod* 1998, **61**:961–964.

106. Meyer JJM, Rakuambo NC, Hussein AA: **Novel xanthones from Securidaca longepedunculata with activity against erectile dysfunction**. *J Ethnopharmacol* 2008, **119**:599–603.

107. Mimaki Y, Kuroda M, Yokosuka A, Sashida Y: **Five new polyoxygenated cholestane bisdesmosides from the bulbs of Galtonia candicans**. *J Nat Prod* 2001, **64**:1069–1072.

108. Mimaki Y, Kuroda M, Kameyama A, Sashida Y, Hirano T, Oka K, Dobashi A, Koike K, Nikaido T: **A new rearranged cholestane glycoside from Ornithogalum saundersiae bulbs exhibiting potent cytostatic activities on leukemia HL-60 and MOLT-4 cells**. *Bioorganic Med Chem Lett* 1996, **6**:2635–2638.

109. Mimaki Y, Kuroda M, Kameyama A, Sashida Y, Hirano T, Oka K, Dobashi A, Koike K, Nikaido T: **A new rearranged cholestane glycoside from Ornithogalum saundersiae bulbs exhibiting potent cytostatic activities on leukemia HL-60 and MOLT-4 cells**. *Tetrahedron Lett* 1996, **6**:2635–2638.

110. Mimaki Y, Kuroda M, Kameyama A, Sashida Y, Hirano T, Oka K, Maekawa R, Wada T, Sugita K, Beutler JA: **Cholestane glycosides with potent cytostatic activities on various tumor cells from Ornithogalum saundersiae bulbs**. *Bioorganic Med Chem Lett* 1997, **7**:633–636.

111. Mnonopi N, Levendal RA, Davies-Coleman MT, Frost CL: **The cardioprotective effects of marrubiin, a diterpenoid found in Leonotis leonurus extracts**. *J Ethnopharmacol* 2011, **138**:67–75.

112. Moser BR: **Review of cytotoxic cephalostatins and ritterazines: Isolation and synthesis**. *J Nat Prod* 2008, **71**:487–491.

113. Mulholland DA, Koorbanally C, Crouch NR, Sandor P: **Xanthones from Drimiopsis maculata**. *J Nat Prod* 2004, **67**:1726–1728.

114. Munkombwe NM, Maswabi T, Hughes NA: **Diosphenols from Spirostachys africana**. *Phytochemistry* 1997, **45**:1217–1220.

115. Nair JJ, Campbell WE, Brun R, Viladomat F, Codina C, Bastida J: **Alkaloids from Nerine filifolia**. *Phytochemistry* 2005, **66**:373–382.

116. Neergaard JS, Andersen J, Pedersen ME, Stafford GI, Staden J Van, Jäger AK: **Alkaloids from Boophone disticha with affinity to the serotonin transporter**. *South African J Bot* 2009, **75**:371–374.

117. Nyila MA, Leonard CM, Hussein AA, Lall N: **Activity of South African medicinal plants against Listeria monocytogenes biofilms, and isolation of active compounds from Acacia karroo**. *South African J Bot* 2012, **78**:220–227.

118. Omoruyi BE, Afolayan AJ, Bradley G: **The inhibitory effect of Mesembryanthemum edule (L.) bolus essential oil on some pathogenic fungal isolates.** *BMC Complement Altern Med* 2014, **14**:168.

119. Orsini F, Pelizzoni F, Verotta L, Aburjai T, Rogers CB: **Isolation, synthesis, and antiplatelet aggregation activity of resveratrol 3-O-??-D-glucopyranoside and related compounds**. *J Nat Prod* 1997, **60**:1082–1087.

120. Patil AD, Freyer AJ, Breen A, Carte B, Johnson RK: **Halistanol disulfate B, a novel sulfated sterol from the sponge Pachastrella sp.: Inhibitor of endothelin converting enzyme**. *J Nat Prod* 1996, **59**:606–608.

121. Patil AD, Freyer AJ, Killmer L, Zuber G, Carte B, Jurewicz AJ, Johnson RK: **Sp.: Inhibitor of Interleukin-8 Receptors**. *Nat Prod Lett* 1997, **10**(March 2015):225–229.

122. Pedersen MM, Chukwujekwu JC, Lategan CA, Staden J Van, Smith PJ, Staerk D: **Antimalarial sesquiterpene lactones from Distephanus angulifolius**. *Phytochemistry* 2009, **70**:601–607.

123. Pendota SC, Aderogba MA, Ndhlala AR, Van Staden J: **Antimicrobial and acetylcholinesterase inhibitory activities of Buddleja salviifolia (L.) Lam. leaf extracts and isolated compounds**. *J Ethnopharmacol* 2013, **148**:515–520.

124. Perold GW: **9. The structure of geigerin**. *J Chem Soc* 1957:47.

125. Pettit GR, Xu JP, Williams MD, Christie ND, Doubek DL, Schmidt JM, Boyd MR: **Isolation and structure of cephalostatins 10 and 11.** *J Nat Prod* 1994, **57**:52–63.

126. Pettit GR, Herald CL, Cichacz ZA, Gao F, Schmidt JM, Boyd MR, Christie ND, Boettner FE: **Isolation and structure of the powerful human cancer cell growth inhibitors spongistatins 4 and 5 from an African Spirastrella spinispirulifera(porifera)**. *J Chem Soc Chem Commun* 1993, **03**:1805.

127. Pettit GR, Inoue M, Kamano Y, Dufresne C, Christie N, Niven ML, Herald DL: **Isolation and structure of the hemichordate cell growth inhibitors cephalostatins 2, 3, and 4**. *J Chem Soc Chem Commun* 1988, **3**(E 13700):865.

128. Pettit GR, Kamano Y, Dufresne C, Inoue M, Christie N, Schmidt JM, Doubek DL: **Isolation and structure of the unusual Indian Ocean Cephalodiscus gilchristi components, cephalostatins 5 and 6**. *Can J Chem* 1989, **67**:1509–1513.

129. Pettit GR, Tan R, Xu JP, Ichihara Y, Williams MD, Boyd MR: **Antineoplastic agents. 398. Isolation and structure elucidation of cephalostatins 18 and 19**. *J Nat Prod* 1998, **61**:955–958.

130. Pettit GR, Xu J, Ichihara Y, Williams MD, Boyd MR: **Antineoplastic agents 285. Isolation and structures of cephalostatins 14 and 15**. *Can J Chem* 1994, **72**:2260–2267.

131. Pettit GR, Ichihara Y, Xu J, Boyd MR, Williams MD: **Isolation and structure of the symmetrical disteroidal alkaloids cephalostatin 12 and cephalostatin 13**. *Bioorg Med Chem Lett* 1994, **4**:1507–1512.

132. Pettit GR, Inoue M, Kamano Y, Herald DL, Arm C, Dufresne C, Christie ND, Schmidt JM, Doubek DL, Krupa TS: **Isolation and structure of the powerful cell growth inhibitor cephaplostatin 1**. *J Am Chem Soc* 1988, **110**:2006–2007.

133. Pika J, John Faulkner D: **Unusual chlorinated homo-diterpenes from the South African nudibranch Chromodoris hamiltoni**. *Tetrahedron* 1995, **51**:8189–8198.

134. Pika J, Faulkner DJ: **Four sesquiterpenes from the South African nudibranch Leminda millecra**. *Tetrahedron* 1994, **50**:3065–3070.

135. Popplewell WL, Marais EA, Brand L, Harvey BH, Davies-Coleman MT: **Euphorbias of South Africa: Two New Phorbol Esters from Euphorbia bothae**. *South African J Chem Tydskr Vir Chemie* 2010, **63**:175–179.

136. Prinsloo G, Meyer JJM, Hussein AA, Munoz E, Sanchez R: **A cardiac glucoside with in vitro anti-HIV activity isolated from Elaeodendron croceum.** *Nat Prod Res* 2010, **24**(July 2014):1743–1746.

137. Rabe T, Mullholland D, Van Staden J: **Isolation and identification of antibacterial compounds from Vernonia colorata leaves**. *J Ethnopharmacol* 2002, **80**:91–94.

138. Rabe T, Van Staden J: **Isolation of an antibacterial sesquiterpenoid from Warburgia salutaris**. *J Ethnopharmacol* 2000, **73**:171–174.

139. Rashid MA, Gustafson KR, Cartner LK, Pannell LK, Boyd MR: **New nitrogenous constituents from the South African marine Ascidian Pseudodistoma sp.** *Tetrahedron* 2001, **57**:5751–5755.

140. Reyes F, Ardá A, Martín R, Fernández R, Rueda A, Montalvo D, Gómez C, Jiménez C, Rodríguez J, Sánchez-Puelles JM: **New cytotoxic cembranes from the sea pen Gyrophyllum sibogae**. *J Nat Prod* 2004, **67**(Scheme 1):1190–1192.

141. Rudi A, Schleyer M, Kashman Y: **Clathculins A and B, two novel nitrogen-containing metabolites from the sponge Clathrina aff. reticulum**. *J Nat Prod* 2000, **63**:1434–1436.

142. Rudi A, Kashman Y, Benayahu Y, Schleyer M: **Amino acid derivatives from the marine sponge Jaspis digonoxea.** *J Nat Prod* 1994, **57**:829–832.

143. Rudi A, Yosief T, Schleyer M, Kashman Y: **Several new isoprenoids from two marine sponges of the family Axinellidae**. *Tetrahedron* 1999, **55**:5555–5566.

144. Sandager M, Nielsen ND, Stafford GI, Van Staden J, Jäger AK: **Alkaloids from Boophane disticha with affinity to the serotonin transporter in rat brain**. *J Ethnopharmacol* 2005, **98**:367–370.

145. San-Martín A, Quezada E, Soto P, Rovirosa J, Palacios Y: **Labdane diterpenes from the marine pulmonate gastropod Trimusculus peruvianus**. *Can J Chem* 1996, **74**:2471–2475.

146. Scheepers BA, Klein R, Davies-Coleman MT: **Synthesis of triprenylated toluquinone and toluhydroquinone metabolites from a marine-derived Penicillium fungus**. *Tetrahedron Lett* 2006, **47**:8243–8246.

147. Sehlapelo BM, Drewes SE, Sandor P: **Ocobullenone: A bicyclo[3.2.1]octanoid neolignan from Ocotea bullata**. *Phytochemistry* 1993, **32**:1352–1353.

148. Shode FO, Mahomed AS, Rogers CB: **Typhaphthalide and typharin, two phenolic compounds from Typha capensis**. *Phytochemistry* 2002, **61**:955–957.

149. Sibanda S, Nyanyira C, Nicoletti M, Galeffi C: **Vismiones L and M from Ochna pulchra**. *Phytochemistry* 1993, **34**:1650–1652.

150. Sidwell WTL, Fritz H, Tamm C: **Autumnariol und Autumnariniol, zwei neue Dibenzo-α-pyrone aus Eucomis autumnalis Graeb. Nachweis einer Fernkopplung über sechs Bindungen in den magnetischen Protonenresonanz - Spektren**. *Helv Chim Acta* 1971, **54**:207–215.

151. Sidwell WTL, Tamm C: **Errata: The homo-isoflavones II1). Isolation and structure of 4’-o-methyl-punctatin, autumnalin and 3,9-dihydro-autumnalin**. *Tetrahedron Lett* 1970, **11**:1578.

152. Sikorska J, Hau AM, Anklin C, Parker-Nance S, Davies-Coleman MT, Ishmael JE, McPhail KL: **Mandelalides A-D, cytotoxic macrolides from a new Lissoclinum species of South African tunicate**. *J Org Chem* 2012, **77**:6066–6075.

153. Sikorska J, Parker-Nance S, Davies-Coleman MT, Vining OB, Sikora AE, McPhail KL: **Antimicrobial rubrolides from a South African species of synoicum tunicate**. *J Nat Prod* 2012, **75**:1824–1827.

154. Sunassee SN, Davies-Coleman MT: **Cytotoxic and antioxidant marine prenylated quinones and hydroquinones**. *Nat Prod Rep* 2012, **29**:513.

155. Tarus PK, Coombes PH, Crouch NR, Mulholland DA, Moodley B: **Furoquinoline alkaloids from the southern African Rutaceae Teclea natalensis**. *Phytochemistry* 2005, **66**:703–706.

156. Van der Kooy F, Meyer JJM, Lall N: **Antimycobacterial activity and possible mode of action of newly isolated neodiospyrin and other naphthoquinones from Euclea natalensis**. *South African J Bot* 2006, **72**:349–352.

157. Van Heerden FR, Viljoen AM, van Wyk BE: **The major flavonoid of Dodonaea angustifolia.** *Fitoterapia* 2000, **71**:602–604.

158. Van Heerden FR, Marthinus Horak R, Maharaj VJ, Vleggaar R, Senabe J V., Gunning PJ: **An appetite suppressant from Hoodia species**. *Phytochemistry* 2007, **68**:2545–2553.

159. Van Heerden FR, Viljoen AM, Van Wyk BE: **6’-O-coumaroylaloesin from Aloe castanea - A taxonomic marker for Aloe section Anguialoe**. *Phytochemistry* 2000, **55**:117–120.

160. Van Wyk AWW, Davies-Coleman MT: **Semisynthesis of labdane diterpene metabolites from the nudibranch Pleurobranchaea meckelii**. *Tetrahedron* 2007, **63**:12179–12184.

161. Van Wyk AWW, Gray CA, Keyzers RA, Rivett DEA, Caira MR, Nader BS, Davis GE, Werk TL, Davies-Coleman MT: **Transformations of hispanolone. Novel Michael adducts with in planta activity against rice blast**. *Tetrahedron* 2005, **61**:8493–8498.

162. Van Wyk AWW, Gray CA, Whibley CE, Osoniyi O, Hendricks DT, Caira MR, Davies-Coleman MT: **Bioactive metabolites from the South African marine mollusk Trimusculus costatus**. *J Nat Prod* 2008, **71**:420–425.

163. Van Zyl RL, Khan F, Edwards TJ, Drewes SE: **Antiplasmodial activities of some abietane diterpenes from the leaves of five Plectranthus species**. *S Afr J Sci* 2008, **104**(February):62–64.

164. Verotta L, Orsini F, Pelizzoni F, Torri G, Rogers CB: **Polyphenolic glycosides from african proteaceae**. *J Nat Prod* 1999, **62**:1526–1531.

165. Weigenand O, Hussein AA, Lall N, Meyer JJM: **Antibacterial activity of naphthoquinones and triterpenoids from Euclea natalensis root bark**. *J Nat Prod* 2004, **67**:1936–1938.

166. Whibley CE, Keyzers RA, Soper AG, Davies-Coleman MT, Samaai T, Hendricks DT: **Antiesophageal cancer activity from Southern African marine organisms**. *Ann N Y Acad Sci* 2005, **1056**:405–412.

167. Whibley CE, McPhail KL, Keyzers RA, Maritz MF, Leaner VD, Birrer MJ, Davies-Coleman MT, Hendricks DT: **Reactive oxygen species mediated apoptosis of esophageal cancer cells induced by marine triprenyl toluquinones and toluhydroquinones.** *Mol Cancer Ther* 2007, **6**:2535–2543.

168. Yeboah EMO, Majinda RRT, Kadziola A, Muller A: **Dihydro-??-agarofuran sesquiterpenes and pentacyclic triterpenoids from the root bark of Osyris lanceolata**. *J Nat Prod* 2010, **73**:1151–1155.

169. Yelani T, Hussein AA, Meyer JJM: **Isolation and identification of poisonous triterpenoids from Elaeodendron croceum.** *Nat Prod Res* 2010, **24**(July 2014):1418–1425.

170. Zoraghi R, Worrall L, See RH, Strangman W, Popplewell WL, Gong H, Samaai T, Swayze RD, Kaur S, Vuckovic M, Finlay BB, Brunham RC, McMaster WR, Davies-Coleman MT, Strynadka NC, Andersen RJ, Reiner NE: **Methicillin-resistant Staphylococcus aureus (MRSA) pyruvate kinase as a target for bis-indole alkaloids with antibacterial activities**. *J Biol Chem* 2011, **286**:44716–44725.
