# Supplementary material for: SANCDB: a South African natural compound database
Source: J Cheminform. 2015 Jun 19;7:29. doi: 10.1186/s13321-015-0080-8 (PMC4471313; doi:10.1186/s13321-015-0080-8)
Supplement: Additional file 4: — S-Data 4. Structural classifications of compounds within SANCDB, by source organism and by use. A) The most common compound classifications found in specific source organisms are tabulated. B) The most common compound classifications that were associated with a specific use. Classifications were included in each table if they related to five or more compounds associated with the source or use. [file 13321_2015_80_MOESM4_ESM.docx]

| **Classification** | **Organism** | **No. of Compounds** |
| --- | --- | --- |
| Cholestane | *Ornithogalum* species | 28 |
|  | *Galtonia candicans* | 11 |
| Cephalostatin | *Cephalodiscus gilchristi* | 18 |
|  | *Solanum* species | 2 |
| Cembranolide | *Croton gratissimus* | 12 |
| Sodwanone | *Axinella sppecies* | 8 |
| Naphthoquinone | *Euclea natalensis* | 7 |
| Pyrano-isoflavone | *Eriosema kraussianum* | 7 |
| Polypropionate | *Siphonaria species* | 6 |
| Cyclic peptide | *Jaspis digonoxea* | 6 |
| Rubrolide Furanone | *Synoicum globosum* | 6 |
| Aporphine | *Cissampelos capensis* | 6 |
| Saponins | *Ornithogalum thyrsoides* | 5 |
| Tsitsikammamine | *Tsitsikamma species* | 5 |
| Hamiltonin | *Chromodoris hamiltoni* | 5 |
| Bisdesmoside | *Galtonia candicans* | 5 |
| Spirostanol | *Ornithogalum thyrsoides* | 5 |

| **Classification** | **Use** | **No. of Compounds** |
| --- | --- | --- |
| Cephalostatin | Anticancer | 19 |
| Naphthoquinone | Antibacterial | 6 |
| Polyketide | Anticancer | 6 |
| Rubrolide Furanone | Antibacterial | 5 |
