# Supplementary figures and images for: SANCDB: a South African natural compound database
Source: J Cheminform. 2015 Jun 19;7:29. doi: 10.1186/s13321-015-0080-8 (PMC4471313; doi:10.1186/s13321-015-0080-8)

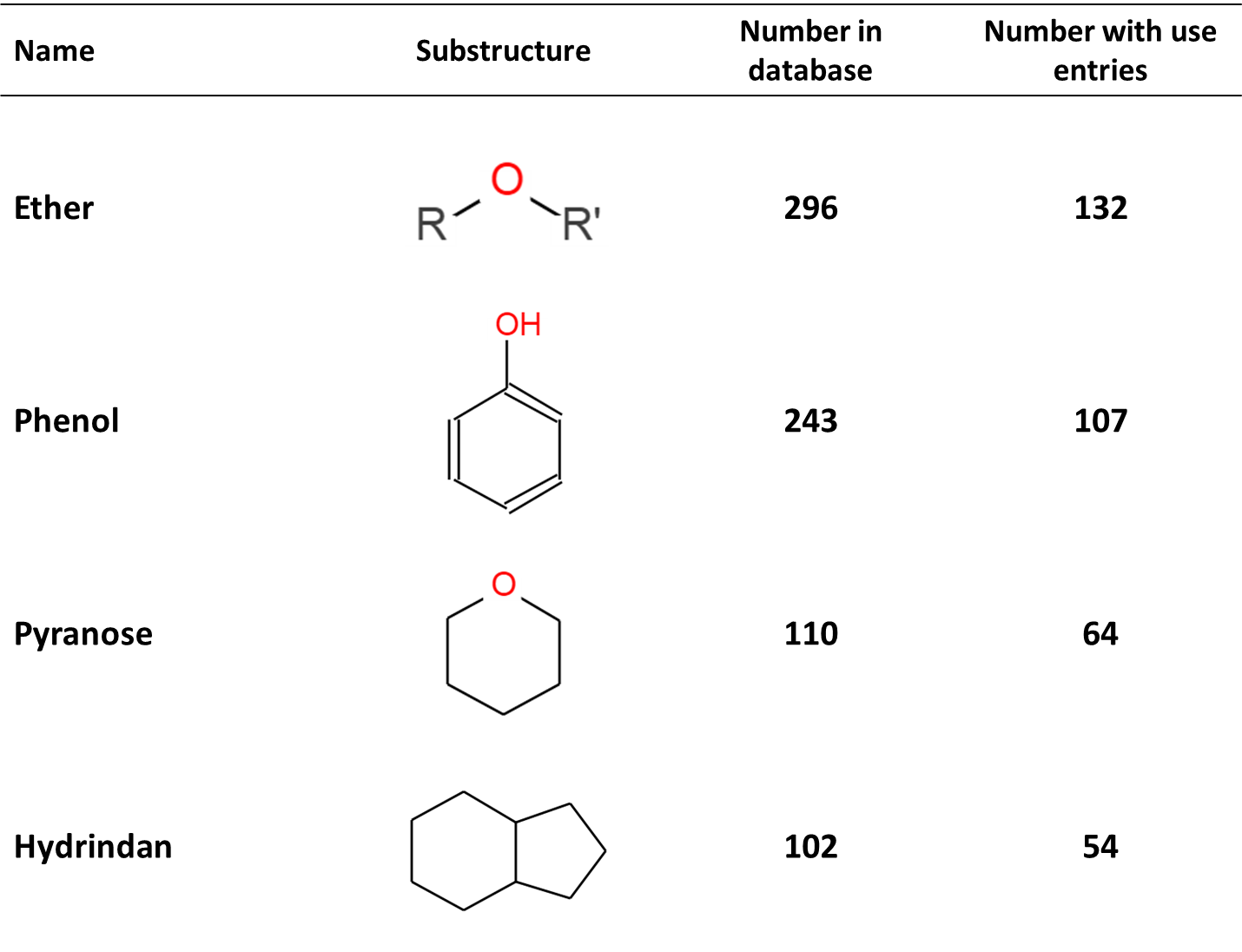


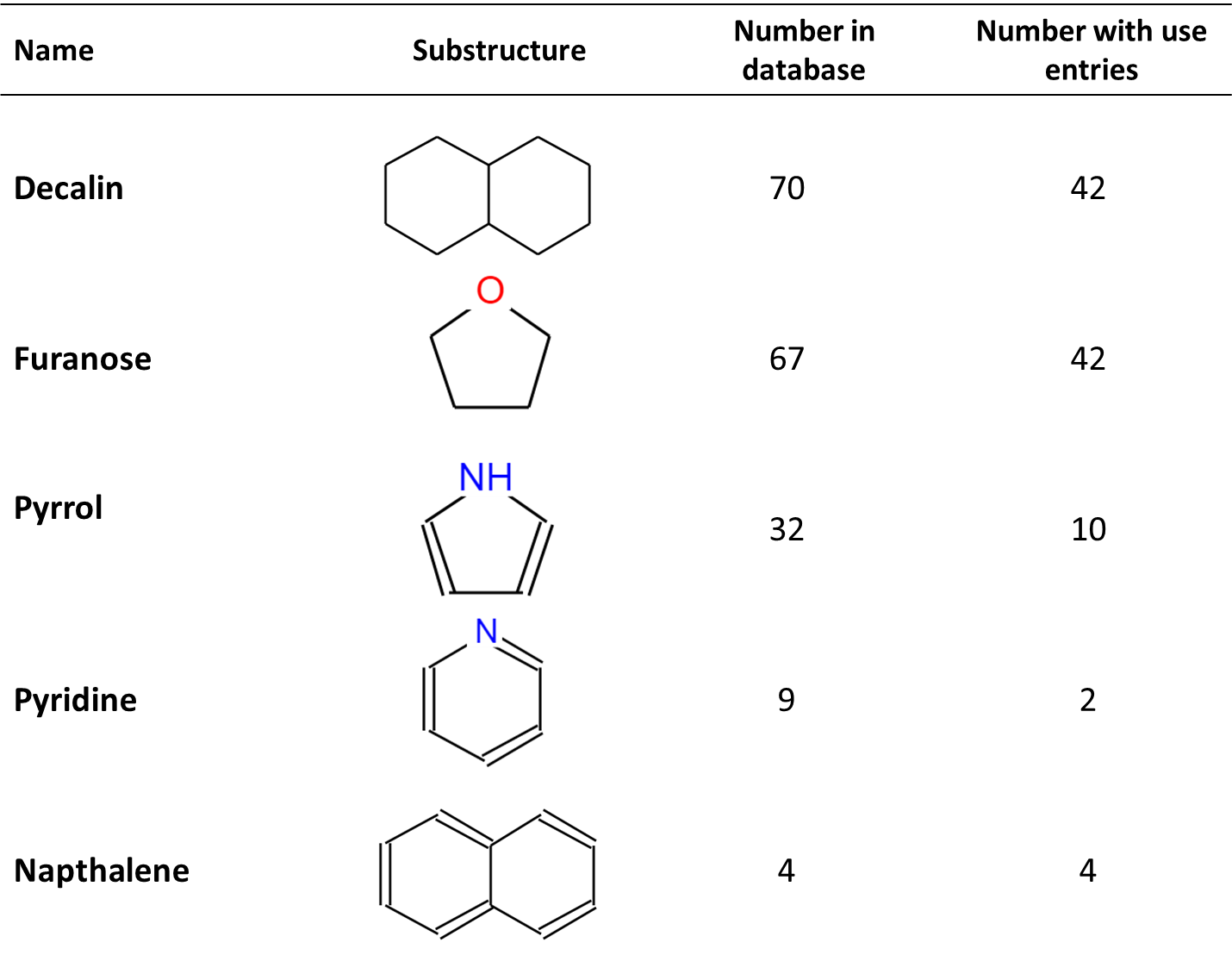

Supplement: Additional file 5: — S-Data 5. Structural motifs of compounds within SANCDB. The names and structures of specific motifs present in the compounds in SANCDB are shown. Next to this is the number of compounds in the database which contain the structural motif, as well as the number of these compounds with recorded uses in SANCDB. [file 13321_2015_80_MOESM5_ESM.docx]
